# Endoscopic Management of a Case of Spontaneous Cerebrospinal Fluid Leaks in Anterior Skull Base

**DOI:** 10.7759/cureus.62042

**Published:** 2024-06-10

**Authors:** Tomoharu Suzuki, Noritaka Komune, Yusuke Miyamoto, Daisuke Murakami, Takashi Nakagawa

**Affiliations:** 1 Otolaryngology - Head and Neck Surgery, Kyushu University, Fukuoka, JPN; 2 Medical Sciences, Kyushu University, Fukuoka, JPN; 3 Otolaryngology - Head and Neck Surgery, Kyushu University Hospital, Fukuoka, JPN

**Keywords:** nasal mucosal flap, endoscopic transnasal approach, csf, cribriform plate, spontaneous cerebrospinal fluid rhinorrhea

## Abstract

Spontaneous cerebrospinal fluid (sCSF) leaks are rare, and their diagnosis and treatment often present significant challenges. This paper discusses and reports cases experienced at our facility. We retrospectively reviewed three of five cases of sCSF leaks experienced at the Department of Otolaryngology and Head and Neck Surgery, Kyushu University, from December 2020 to December 2022, excluding CSF otorrhea. All three patients were female; their mean age was 56 years (44-71 years). Two of the three patients were obese (first degree), and one was average weight (according to the criteria of the Japan Society for the Study of Obesity). Two patients had hypertension, and one had sleep apnea syndrome as an underlying disease. In all cases, leakage sites, which were all the cribriform plate, can be endoscopically identified, and all could be closed by an endoscopic intranasal approach. We reviewed cases of sCSF leaks. Although some patients had difficulty identifying the leakage site in a narrow and complex nasal cavity, an endoscopic survey was useful in identifying the leakage site. All cases were closed and there were no signs of recurrence. Identifying the site of leakage and selecting the appropriate closure method depending on the extent of the leakage is essential in treating such cases.

## Introduction

Cerebrospinal fluid (CSF) rhinorrhea can be categorized into congenital and acquired types. Acquired CSF rhinorrhea is further subdivided into traumatic (head injury, surgical trauma, etc.) and non-traumatic (tumor, infection, etc.) forms, with cases of unknown origin referred to as idiopathic CSF rhinorrhea. Among acquired CSF rhinorrhea cases, over 80% are, while spontaneous cerebrospinal fluid (sCSF) leaks are relatively rare. Idiopathic intracranial hypertension (IIH) is known to have a high incidence of association, and it is hypothesized that prolonged elevation of intracranial pressure can lead to thinning and destruction of the skull base bones, eventually resulting in sCSF leaks [1]. Effective management of sCSF leaks in the nasal cavity requires accurate detection and precise leak localization [2]. From December 2020 to December 2022, we treated three cases of sCSF leaks in the anterior skull base at our institution. In this report, we present these cases and discuss the strategies for managing sCSF leaks in anterior skull bases. We retrospectively reviewed three cases of spontaneous CSF rhinorrhea out of five cases treated at the Department of Otorhinolaryngology and Head and Neck Surgery, Kyushu University, from December 2020 to December 2022. The two excluded cases were both CSF otorrhea.

## Case presentation

Results

Case Profiles

All three patients were female, with a mean age of 56 years (44-71). Two of the three patients were categorized as obese (first degree), and one was average weight (according to the criteria of the Japan Society for the Study of Obesity) [3].

Two patients had hypertension, and one had sleep apnea syndrome as an underlying condition. In all cases, the leakage site was from the cribriform plate, and all could be closed using an endoscopic intranasal approach. One case was repaired with free nasal septal mucosa, and two patients with fat were taken from the groin (Table 1).

**Table 1:** Case profiles CP: cribriform plate, HT: hypertension, MT: middle turbinate, NSM: nasal septum mucosa, SAS: sleep apnea syndrome, ST: superior turbinate

#	Sex	Age	BMI	Location	Medical history	Filling tissue	Flap (P=pedicle, F=free)
1	F	71	24.4	CP	HT	NSM	NSM (F)
2	F	53	26.8	CP	HT, SAS	Fat	MT (P)
3	F	44	25	CP	None	Fat	NSM (P), ST (F)

Illustrative Cases (Case 1)

**Figure 1:**
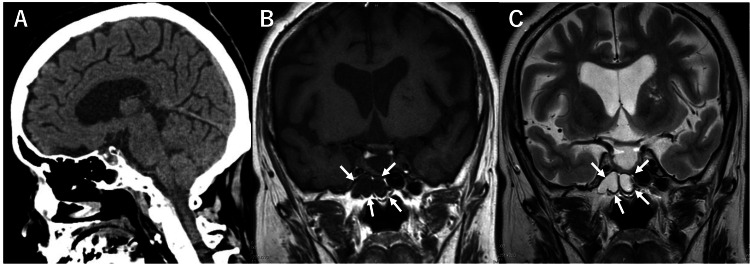
Preoperative CT and MRI findings (case 1) (A) Preoperative sagittal CT showed no obvious bone defects at the skull base. (B) The coronal T1-weighted MRI image showed a low signal within the sphenoid sinus. In contrast (C), the coronal T2-weighted MRI image showed a high signal, suggesting the presence of fluid accumulation within the sphenoid sinus (white arrow). CT, computed tomography; MRI, magnetic resonance imaging

**Figure 2:**
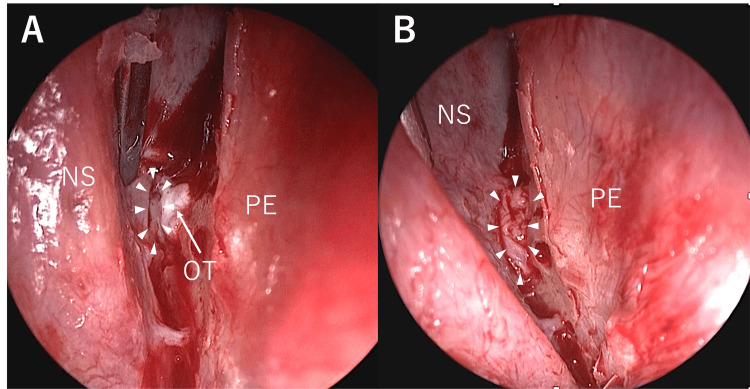
A site of leakage (A) A site of leakage (white arrow) was identified after the excision of a strand of the olfactory nerve. (B) The excised nasal septal mucosa was covered over the leakage site, and the cessation of CSF leakage was confirmed. NS, nasal septum; OT, olfactory thread; PE, perpendicular plate of the ethmoid bone; CSF, cerebrospinal fluid

A 72-year-old woman presented with a right-sided nasal discharge for one month and was initially seen at a nearby otorhinolaryngology clinic. She had no neurological abnormalities, and there were no signs suggestive of meningitis. There was no history of surgery, trauma, or allergies. Glucose was detected in the nasal discharge, leading to the diagnosis of CSF rhinorrhea. Physical examination revealed a watery nasal discharge from the right nostril without fever or signs of meningeal irritation. The patient had no history of trauma, but she had a medical history of hypertension and Parkinson's syndrome. She was referred to our hospital, and it was decided to perform a joint operation involving the otorhinolaryngology and neurosurgery departments. The glucose test from nasal discharge was still positive at our hospital. Although computed tomography (CT) and magnetic resonance imaging (MRI) did not show any obvious bone defects, a high-intensity area on the T2-weighted MRI image was observed within the sphenoid sinus (Figure 1A-1C). Suspecting CSF leaks from the sphenoid sinus, we performed an endoscopic endonasal surgery. The fluid was observed coming out of the natural ostium of the sphenoid sinus. Despite the extensive opening of the area, the exact leak site within the sphenoid sinus could not be identified. Upon careful examination, dissection of the nasal septum, and removal of an olfactory filament, we could identify the site of the leak on the cribriform plate (Figure 2A). Given the small size of the leakage site, we inserted the nasal septal mucosa covered over the leakage site and performed packing, then completed the procedure (Figure 2B). There has been no recurrence more than two years after surgery.

Illustrative Cases (Case 2)

**Figure 3:**
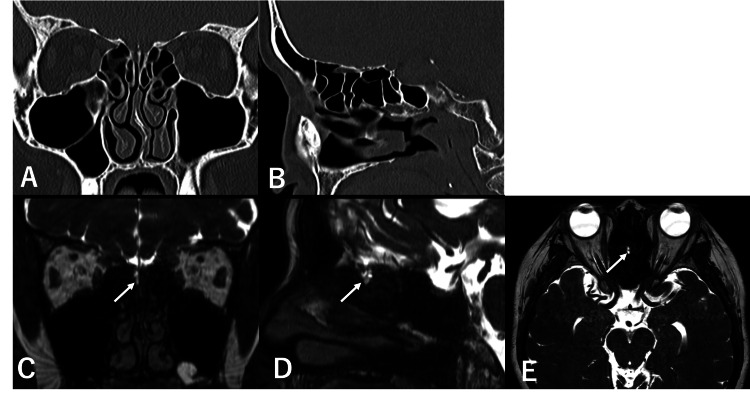
Preoperative CT and MRI findings (case 2) On preoperative (A) coronal and (B) sagittal CT scans, an area on the right cribriform plate appeared to be a bone defect, but this could not be definitively confirmed. (C) Coronal, (D) sagittal, (E) axial MRI CISS showed a high signal on the right cribriform plate (white arrow). CT, computed tomography; MRI, magnetic resonance imaging; CISS, constructive interference in steady state

The case is a 53-year-old woman who presented to a nearby otorhinolaryngology clinic with a chief complaint of right nasal discharge for two months. Suspecting a sCSF leak, she was referred to our hospital. Physical examination revealed a watery nasal discharge from the right side and a low-grade fever lasting approximately three weeks. There was no sign of meningeal irritation, and meningitis was ruled out. The patient had no history of trauma, but she had a medical history of hypertension and previous sleep apnea. The glucose test from nasal discharge was positive. CT scan indicated a potential bone defect in the right cribriform plate, though this could not be definitively confirmed (Figure 3A, 3B). However, MRI CISS (constructive interference in steady state) showed a high-intensity area on the T2-weighted image on the right cribriform plate (Figure 3C-3E), suggesting CSF leakage from this site.

**Figure 4:**
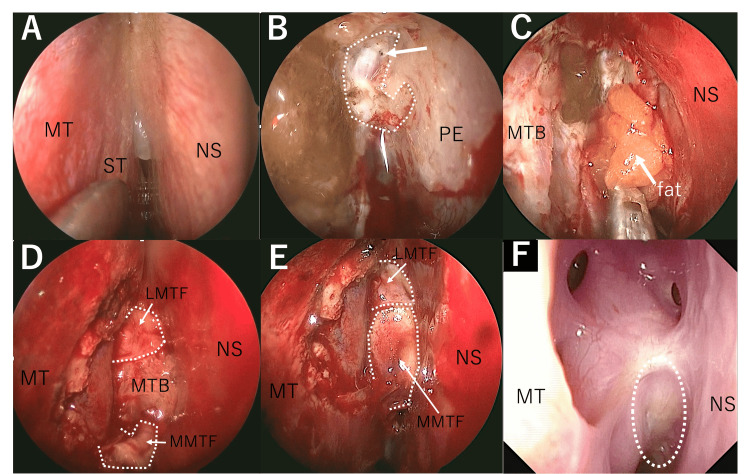
Reconstruction techniques (case 2） (A) The right middle and superior turbinate lateralization revealed an elevated cribriform plate lesion. (B) Dissection of the mucosa anterior to the lesion revealed areas of bone defect (white line), dural defect (white arrow), and CSF rhinorrhea. (C) We took fat from the abdominal area and applied it to the exposed area with fibrin glue (Bolheal®). (D) MTB was placed over the fat and a LMTF was created. (E) Anterior defect covered with LMTF and posterior defect covered with MMTF and used Bolheal® to complete the procedure. (F) Fiberscopy findings at three months postoperatively. A white circle indicates the reconstructed area. LMTF, lateral middle turbinate flap; MT, middle turbinate; MTB, middle turbinate bone; MMTF, medial middle turbinate flap; NS, nasal septum; PE, perpendicular plate of the ethmoid bone; ST, superior turbinate; CSF, cerebrospinal fluid

Based on these findings, the endoscopic endonasal surgery was performed. The right middle and superior turbinate lateralization revealed a bulging area on the cribriform plate lesion (Figure 4A). The mucosa was dissected from the area anterior to that region and we finally confirmed the location of the bone defect and CSF leaks (Figure 4B). The abdominal fat was harvested and inserted into the bony defect area and fixed with fibrin glue (Bolheal®) (Figure 4C). Middle turbinate bone (MTB) was placed over the fat and a regional lateral middle turbinate flap (LMTF) was created (Figure 4D). The anterior defect was covered with LMTF and the posterior defect was covered with a regional medial middle turbinate flap (MMTF) and used Bolheal® to complete the procedure (Figure 4E). There has been no recurrence over a year after surgery (Figure 4F).

## Discussion

Among cases of acquired CSF rhinorrhea, more than 80% are due to traumatic CSF leaks, making sCSF leaks relatively rare [1]. There have also not been a few case reports of sCSF leaks in the anterior skull base. We retrospectively reviewed three cases of sCSF leaks treated at our facility. Consistent with previous reports, all three cases involved female patients, and there was a tendency for the patients to be obese (two out of three cases were obese) [4,5]. sCSF leaks are also known to be associated with a high rate of idiopathic intracranial hypertension (IIH), which is thought to be caused by thinning and destruction of the skull base bone due to prolonged elevated intracranial pressure [1,4,6,7]. Additionally, sleep apnea, which also affected one out of three of our patients, has been suggested to be associated with sCSF leaks [8]. Past reports have indicated that the cribriform plate is often involved as a defect site [5,9]. In a review conducted by Hong et al. involving 716 patients, the sites of sCSF leaks were reported as follows: sphenoid sinus (41.1%), cribriform plate (25.4%), and ethmoid skull base (20.4%) [10]. In our cases, all three of the leaks were detected from the cribriform plate, which is consistent with previous reports. In one of three of our cases, although imaging suggested that the sphenoid sinus was the site of CSF leakage, it ultimately turned out to be the cribriform plate. Therefore, it is essential to thoroughly inspect the cribriform plate during endoscopic endonasal surgery. To diagnose a CSF leak, it is essential to identify the presence of a CSF leak and to localize the leak. The β2 transferrin test is commonly used due to its high sensitivity and specificity for diagnosis [1,2]. In our series, glucose testing was performed in all three cases since β2 transferrin testing can only be available in limited facilities in Japan. While glucose testing is simple, it may yield false-positive results in people with diabetes and other conditions. High-resolution CT (HRCT) scan is the first choice for leakage site detection [2,11,12]. However, in all three cases in this study, the bony defects could not be accurately identified by HRCT. As mentioned above, the HRCT findings suggested a leak in the sphenoid sinus in one of three cases, and surgery was first performed on the sphenoid sinus. This surgical procedure could have been avoided with sufficient endonasal fiberscope observation in the outpatient department and a more accurate preoperative diagnosis. Magnetic resonance cisternography (MRC) is also valuable for identifying the location of leaks, showing communication between the CSF and the extracranial space, even without a meningocele [13]. However, MRC must be interpreted alongside HRCT as MRI does not provide optimal bone detail [14]. For spontaneous CSF leak, the success rate of initial surgery for treatment using the transnasal approach is around 80-90%, which is considered higher than that of craniotomy [1,4,5,7]. Due to its minimally invasive nature, the transnasal approach is considered the first choice for surgery. The endoscope is also useful for diagnosis, allowing detailed intraoperative direct visualization of the leak, as seen in case 1. Although multi-layer repairs are considered the most common repair method, the actual materials used will depend on the size of the skull base defect. Minor defects (<5 mm) are usually closed with free grafts. Other methods for the leaks from the cribriform plate involve cauterizing the leakage site with a bipolar, placing gelfoam, and suturing the middle turbinate to the nasal septum [15].

Typically, cribriform leaks are repaired in two to three layers. There are short-term failure rates of 9% and 6.5%, respectively, and long-term failure rates are low, as this review states [4]. In case 1, the defect was minor, and the nasal septal mucosa was used as a free graft to close the defect. In the other two cases, the defects were larger, filled with fat, and reconstructed with a pedicled flap over the fat graft. In case 2, we performed a multilayered reconstruction with a pedicled rotated mini flap using the middle turbinate. This flap is mainly supplied from the sphenopalatine artery and is also thought to have elements of a random pattern flap with blood flow supplied by the surrounding capillaries. We have named this flap the MMTF, which may be aggressively used for CSF leakage from the cribriform plate. In the near future, increasing the number of cases using this flap and monitoring long-term outcomes are necessary.

## Conclusions

Through these cases, we have reaffirmed that the cribriform plate is a common site of leakage in spontaneous CSF leaks despite the small number of cases. In addition, when it is difficult to identify the leakage site by CT or MRI, appropriate endoscopic observation is necessary. At this point, it is always necessary to assume leakage from the cribriform plate. We have devised a new flap, the MMTF. It appears to be effective for managing CSF leaks originating from the cribriform plate, so we would like to increase the number of cases in the future and conduct further investigations. Additionally, we hope it can serve as a reference for readers performing similar surgeries.

## References

[1] Alonso RC, de la Peña MJ, Caicoya AG, Rodriguez MR, Moreno EA, de Vega Fernandez VM (2013). Spontaneous skull base meningoencephaloceles and cerebrospinal fluid fistulas. Radiographics.

[2] Oakley GM, Alt JA, Schlosser RJ, Harvey RJ, Orlandi RR (2016). Diagnosis of cerebrospinal fluid rhinorrhea: an evidence-based review with recommendations. Int Forum Allergy Rhinol.

[3] Examination Committee of Criteria for 'Obesity Disease' in Japan (2002). New criteria for 'obesity disease' in Japan. Circ J.

[4] Lobo BC, Baumanis MM, Nelson RF (2017). Surgical repair of spontaneous cerebrospinal fluid (CSF) leaks: a systematic review. Laryngoscope Investig Otolaryngol.

[5] Englhard AS, Volgger V, Leunig A, Meßmer CS, Ledderose GJ (2018). Spontaneous nasal cerebrospinal fluid leaks: management of 24 patients over 11 years. Eur Arch Otorhinolaryngol.

[6] Ali M, Elgassim MA, Faisal HM, Saied AS, Elgassim M (2023). Spontaneous cerebrospinal fluid rhinorrhea secondary to idiopathic intracranial hypertension. Cureus.

[7] Martínez-Capoccioni G, Serramito-García R, Martín-Bailón M, García-Allut A, Martín-Martín C (2017). Spontaneous cerebrospinal fluid leaks in the anterior skull base secondary to idiopathic intracranial hypertension. Eur Arch Otorhinolaryngol.

[8] Bakhsheshian J, Hwang MS, Friedman M (2015). Association between obstructive sleep apnea and spontaneous cerebrospinal fluid leaks: a systematic review and meta-analysis. JAMA Otolaryngol Head Neck Surg.

[9] Gharzouli I, Verillaud B, Tran H (2016). Value of systematic analysis of the olfactory cleft in case of cerebrospinal rhinorrhea: incidence of olfactory arachnoid dilatation. Eur Arch Otorhinolaryngol.

[10] Hong CS, Kundishora AJ, Elsamadicy AA, Vining EM, Manes RP, Omay SB (2022). A unique subset: idiopathic intracranial hypertension presenting as spontaneous CSF leak of the anterior skull base. J Neurol Surg B Skull Base.

[11] Manes RP, Ryan MW, Marple BF (2012). A novel finding on computed tomography in the diagnosis and localization of cerebrospinal fluid leaks without a clear bony defect. Int Forum Allergy Rhinol.

[12] Abuabara A (2007). Cerebrospinal fluid rhinorrhoea: diagnosis and management. Med Oral Patol Oral Cir Bucal.

[13] Lloyd KM, DelGaudio JM, Hudgins PA (2008). Imaging of skull base cerebrospinal fluid leaks in adults. Radiology.

[14] Tuntiyatorn L, Laothammatas J (2004). Evaluation of MR cisternography in diagnosis of cerebrospinal fluid fistula. J Med Assoc Thai.

[15] Sasindran V, Mathew N, Shabna AK, Harikrishan B (2018). Spontaneous medial cribriform CSF leak: endoscopic surgical repair with free mucosal graft-our experience. Indian J Otolaryngol Head Neck Surg.

